# Visual Working Memory for Faces and Facial Expressions as a Useful “Tool” for Understanding Social and Affective Cognition

**DOI:** 10.3389/fpsyg.2019.02392

**Published:** 2019-10-22

**Authors:** Filippo Gambarota, Paola Sessa

**Affiliations:** ^1^Department of Developmental Psychology and Socialization, University of Padua, Padua, Italy; ^2^Padova Neuroscience Center, University of Padua, Padua, Italy

**Keywords:** visual working memory, face, facial expressions, social cognition, affective cognition, psychopathology

## Abstract

Visual working memory (VWM) is one of the most investigated cognitive systems functioning as a *hub* between low- and high-level processes. Remarkably, its role in human cognitive architecture makes it a stage of crucial importance for the study of socio-affective cognition, also in relation with psychopathology such as anxiety. Among socio-affective stimuli, faces occupy a place of first importance. How faces and facial expressions are encoded and maintained in VWM is the focus of this review. Within the main theoretical VWM models, we will review research comparing VWM representations of faces and of other classes of stimuli. We will further present previous work investigating if and how both static (i.e., ethnicity, trustworthiness and identity) and changeable (i.e., facial expressions) facial features are represented in VWM. Finally, we will examine research showing qualitative differences in VWM for face representations as a function of psychopathology and personality traits. The findings that we will review are not always coherent with each other, and for this reason we will highlight the main methodological differences as the main source of inconsistency. Finally, we will provide some suggestions for future research in this field in order to foster our understanding of representation of faces in VWM and its potential role in supporting socio-affective cognition.

## Introduction

Faces are processed in a unique fashion starting from initial perceptual stages (i.e., encoding). The domain-specific approach sustains that face processing is carried out in specialized modules (Kanwisher and Yovel, 2006). Contrarily, the domain-general approach considers common mechanisms that may operate on face and non-facial stimuli as well. In this perspective, the main factor leading to different processing for faces compared to non-facial stimuli is the substantial visual expertise for the former (Gauthier et al., 2000). This debate aside, faces seem to be characterized by distinctive processing from early stages and supported by specific brain areas (Haxby et al., 2000, 2002) that may, at least in part, explain how faces are represented in visual working memory (VWM), also when compared to other non-facial stimuli.

VWM is a core cognitive system defined by a limited-space in terms of capacity in which visual information is temporarily stored and manipulated for further processing (Luck, 2008; Liesefeld and Müller, 2019) and in this vein it can be considered as a “form of mental workspace” (Fukuda et al., 2010).

One important dispute regards VWM storage organization in relation to memory item feature (e.g., semantic category, visual complexity, and expertise). When dealing with visually complex items (like Chinese characters, polygons, and faces) a particular class of models seems relevant. *Flexible resource models* (as opposed to *discrete resolution models*; see Luck et al., 1997; Vogel et al., 2001) propose that a limited pool of memory resources can be allocated in a continuous fashion. Each memory representation has a part of noise and the allocation of a larger amount of memory resources leads to less noise and increases item *resolution*. Memory capacity limit occurs because more complex items require a larger amount of resources compared to simpler items (Alvarez and Cavanagh, 2004; Ma et al., 2014; see also Pratte et al., 2017, for a variant of discrete resolution models that consider systematic variation in precision across the stimuli; see also Swan and Wyble, 2014, for an *hybrid model*; see also van den Berg et al., 2012). Differently, *discrete resolution models* (Luck et al., 1997; Vogel et al., 2001) suggest a fixed slot organization of VWM where each memory item is represented within a slot regardless of the feature complexity. Both approaches consider VWM as characterized by limited capacity (3–4 elements on average); however, the concept of complexity is differently treated. Within flexible resource models, the slope in a visual search rate task (i.e., informational load; Alvarez and Cavanagh, 2004) has been proposed as a quantification of visual complexity. In fact, faces are associated with the slowest search rate (i.e., highest *informational load*) and lowest VWM capacity compared to other stimuli (Eng et al., 2005; Jackson and Raymond, 2008; but see Scolari et al., 2008).

Traditionally, VWM has been studied for simple and abstract stimuli (i.e., colored squares, tilted lines) (Luck et al., 1997; Vogel et al., 2001). Nevertheless, a central aspect of human cognition is the processing of stimuli with social and affective content. To note, according to the importance that VWM may have in social and affective cognition, an updated version of Baddeley’s model of working memory (Baddeley and Hitch, 1974) has been more recently proposed considering a specific component devoted to stimuli with emotional content (Baddeley et al., 2012; Xie et al., 2016). Given the importance of VWM in the human cognitive architecture, it is crucial to understand how these emotional stimuli are represented. Among them, faces certainly occupy a place of the highest order. They convey social and affective relevant information such as identity, ethnicity, and emotions.

## Methodological Aspects

For a better comprehension of the studies reviewed in the subsequent sections, this section provides a brief overview on methodological aspects related to VWM research.

One of the traditional paradigms to investigate VWM is the *change detection task* (CDT) (Luck et al., 1997; Vogel et al., 2001; Rensink, 2002). Basically, a *memory array* containing to-be-memorized items is presented, and after a blank *retention interval*, a *test display* is displayed. A behavioral response is needed. Participants are required to compare the to-be-memorized items in the memory array with the item/items presented in the test display. These CDT components roughly correspond to the main VWM operations of encoding, maintenance, and retrieval (Luck, 2008; Liesefeld and Müller, 2019). Although other VWM-related paradigms have also been more or less successfully employed, (e.g., the *n-back* task; Jaeggi et al., 2010), the CDT is the most widely used and is considered the most versatile paradigm for the study of VWM (Luck and Vogel, 2013).

Given the extensive use of this paradigm, this has led to a great proliferation of CDT variants, sometimes at the expense of the interpretation of the results. The most common CDT manipulations regard the amount and/or type of the memory array and test display items, the duration of both the memory array (with a significant impact on the amount of available encoding time for each displayed item) and the retention interval, and the type of test display presented after the retention interval (e.g., single probe vs. whole display; see, e.g., Vogel and Machizawa, 2004; Zhang and Luck, 2008; Brigadoi et al., 2017). One important variant regards the use of a continuous probe display (e.g., choice of a to-be-remembered color from a colors wheel) allowing an estimation of memory precision (Zhang and Luck, 2008; see also Lorenc et al., 2014; Krill et al., 2018 for examples with faces). Other possible variants concern the use of distractors or masks during the retention interval (Vogel et al., 2006).

Within the context of studies that used the CDT, several VWM-dependent measures have been used, including measures of storage capacity (e.g., Cowan’s *K*; Cowan, 2001) – an index of the amount of items effectively retained (for a review on capacity measures, see Rouder et al., 2011) – measures of accuracy – the percentage of correct responses – and measures of sensitivity in the comparison task between the to-be-memorized items and that/those presented in the test display (e.g., *d’* from signal detection theory; Green and Swets, 1974; Wilken and Ma, 2004). As mentioned before, a continuous probe display allows the memory precision estimation through an error distribution around the right value. Finally, the concept of *informational load* (Alvarez and Cavanagh, 2004; Eng et al., 2005) mentioned above is frequently used to compare different stimuli with regard to their visual complexity (but see Jiang et al., 2008).

One of the most studied neural correlate of VWM is an event-related potential (ERP) called *contralateral delay activity* (CDA) or also *sustained posterior contralateral negativity* (SPCN) (for a review, see Luria et al., 2016). This ERP is recorded at occipito-parietal electrodes (*ibidem*) and it has been suggested that the intraparietal sulcus (IPS) is the main neural generator (Xu and Chun, 2006; Robitaille et al., 2009). It is computed as a difference wave (Gratton, 1998) between contralateral and ipsilateral activity related to the hemifield location of to-be-memorized items. CDA amplitude tends to correlate with the amount (Vogel and Machizawa, 2004) and resolution (Luria et al., 2016) of stored visual information and it is also sensitive to visual complexity (colors vs. random polygons; Luria et al., 2010).

Given the great variability in the methods employed and results obtained in the context of VWM studies, we selected those investigations that used a comparable methodology in order to facilitate comparison between results. In some cases, the results of the different studies discussed here are not directly comparable because of differences in the stimuli used (e.g., schematic faces vs. real faces, different facial expressions, etc.) and/or participants’ task (detection of a change in faces identity vs. facial expressions). For this reason, we have tried to indicate details useful to the readers for a critical analysis of the results. In the following sections, we focus on studies using CDT with faces with particular attention to studies that measured the CDA. In the last section of this review, we also discuss studies that considered the relationship between face representations and individual differences (e.g., psychopathology). This review does not aim to be exhaustive but rather to identify and present selected examples of evidence that may help clarify the critical link between VWM functioning and the complexity of social cognition focusing on the main source of social information, that is others’ faces.

## Faces and Visual Working Memory

Curby and Gauthier (2007) demonstrated that a greater number of upright stimuli can be retained in VWM (measured with Cowan’s *K*) compared to inverted ones, and, according to the *face inversion* effect (Yin, 1969; Tanaka and Gordon, 2011), this effect is larger for faces compared to non-facial stimuli (for a review see McKone and Robbins, 2011). Also, the precision is higher for upright faces when compared to inverted faces (Lorenc et al., 2014; Krill et al., 2018). Furthermore, coherent with face visual complexity (Eng et al., 2005; Jiang et al., 2008), this effect is present only if sufficient encoding time (i.e., memory array duration) is provided. One possible explanation for this pattern of results takes into account holistic/configural processing that characterizes faces. In support of this, similar VWM advantage has been reported in expert individuals with other class of objects (Curby et al., 2009; but see Wong et al., 2008; Jiang et al., 2016) or famous faces (Jackson and Raymond, 2008). Within the theoretical framework considering the dissociation between capacity, in terms of slots, and resolution of VWM representations (Scolari et al., 2008; Zhang and Luck, 2008), it has been suggested that perceptual expertise may enhance the resolution of VWM representations (Scolari et al., 2008; Curby and Gauthier, 2010; Lorenc et al., 2014). These results are noteworthy as they strongly suggest that resolution may be a particularly flexible aspect of VWM and potentially modulated on the basis of factors such as, in this case, perceptual expertise, but possibly also social and emotional salience. Therefore, VWM resolution could be a key element for understanding VWM representations of faces and facial expressions of emotions.

### Static and Changeable Facial Features

Faces are characterized by both static and changeable features that convey social and affective information, such as race, identity, trustworthiness (Oosterhof and Todorov, 2009), facial expressions, and gaze direction (Adolphs and Birmingham, 2011).

Recognizing people’s identity is a fundamental social ability (Bruce and Young, 1986; Haxby et al., 2000) and it has been suggested familiarity with specific individual faces might affect their storage in VWM. For this reason, face familiarity could influence VWM in real-time identity processing. Jackson and Raymond (2008) using an identity CDT demonstrated a VWM improvement (capacity and sensitivity) for familiar actors’ faces compared to unfamiliar ones, leading to the conclusion of an involvement of long-term memory in VWM representations of familiar faces. The effect disappeared for inverted faces. Testing pictorial details’ representations of different pictures of the same individual – either familiar or unfamiliar – Dunn et al. (2019, see exp. 2–3) found no difference in performance (in terms of sensitivity) as a function of familiarity.

Race seems to influence the quality of face processing (Young et al., 2012) possibly influencing VWM representations. Zhou et al. (2018) demonstrated that with short encoding time, other-race faces are retained with reduced precision (i.e., standard deviation of errors distributions) compared to own-race faces. Stelter and Degner (2018) demonstrated both lower accuracy (*d’*) and capacity (Cowan’s *K*) for other-race faces. These findings suggest that, similar to inverted faces, other-race faces are processed, at both configural and featural levels of processing, less efficiently (Hayward et al., 2013; Stelter and Degner, 2018). Holistic/configural processing seems a critical aspect in race processing (Tanaka et al., 2004), that may also depend on other social-cognitive factors linked to intergroup processing (for a review, see Young et al., 2012). Interestingly, a previous study has also provided evidence of a reduced CDA amplitude for other-race faces, especially with direct gaze (Sessa and Dalmaso, 2016) and another study reported a correlation between CDA amplitude and implicit racial prejudice scores (Sessa et al., 2012), such that the most prejudiced participants memorized other-race faces with the lowest resolution.

Facial expressions are extremely relevant to social cognition. Information on the others’ affective states (e.g., others’ emotions) and on the environment (e.g., dangers from fearful reactions) could be extracted from facial expressions (Adolphs, 2002). Using similar methodology (i.e., a single-probe identity CDT with real faces; facial expression was task-irrelevant), one recurring finding in VWM literature is that of an advantage in terms of capacity (Cowan’s *K*) and sensitivity (*d’*) for negative facial expressions, especially angry, compared to happy and neutral expressions (Jackson et al., 2008, 2009, 2014; Thomas et al., 2014).

Furthermore, this benefit is observed only when angry faces are presented in the memory array but not in the test display (Jackson et al., 2014). In addition, it declines during the retention interval. Using a longer retention interval (i.e., 9,000 vs. 1,000 ms in the study by Jackson et al., 2009) this benefit disappears (Jackson et al., 2012). Notably, this angry benefit occurs without reducing performance for concurrently presented neutral faces. All stimuli are retained, with an increased resolution for salient stimuli (Thomas et al., 2014). However, slightly different results (i.e., the absence of an angry benefit and/or the presence of an happy benefit) have been reported using schematic facial expressions (i.e., no information on identity), shorter encoding times, or other different methodological details (Langeslag et al., 2009; Simione et al., 2014; Xie et al., 2016; Spotorno et al., 2018; Curby et al., 2019). In particular, the angry face advantage has not always been observed (see also Curby et al., 2019 using a change localization task; Xie et al., 2016 using schematic faces) or has been reported only for short encoding times (150 vs. 1,000/2,000 ms of the previously cited studies) (Simione et al., 2014 using schematic faces).

Varying memory array size, encoding time, and expression (fearful, happy, angry, and neutral), Curby et al. (2019) demonstrated a VWM “cost” for fearful, compared to neutral and happy real faces in terms of lower capacity (Cowan’s *K*). Opposite to the angry benefit, a cost for angry faces has been also observed (Curby et al., 2019) when compared to happy faces (indeed a happy benefit emerged). To note, other studies have instead demonstrated a fearful advantage in terms of capacity, accuracy, and CDA amplitude (Sessa et al., 2011; Stout et al., 2013; Lee and Cho, 2019; all studies used real faces and facial expression was task-irrelevant). Methodological differences could at least in part explain these inconsistent findings. Sessa et al. (2011) and Stout et al. (2013) used a shorter encoding time (200–500) and a smaller set size (1–2) when compared to the study by (Curby et al., 2019; 1,000/4,000 ms and five items, respectively) and the spatial information was less relevant (i.e., the location was probed in Curby et al., 2019). Interestingly, in Curby et al.’s (2019) study, the fear cost emerged only at the longest encoding time and, as argued by authors, a difficulty in disengaging from fearful faces could explain the lower estimated capacity. When controlling for spatial and temporal attention, a fearful advantage in terms of sensitivity (*d’*) emerges (Lee and Cho, 2019).

Overall, the angry face benefit seems consistent across studies. However, changing some CDT parameters like probing method (i.e., probed location), using real vs. schematic faces, different encoding times and/or dependent variables (Cowan *K* vs. *d’*) seems to influence this effect (Langeslag et al., 2009; Simione et al., 2014; Xie et al., 2016; Spotorno et al., 2018; Curby et al., 2019). Similarly, a fearful advantage, relative to neutral faces, is observed for studies using similar parameters (Sessa et al., 2011; Stout et al., 2013; Lee and Cho, 2019; but see Curby et al., 2019). Importantly, CDA seems to differentiate fearful and neutral faces regardless of set size and spatial or temporal attentional biases (Sessa et al., 2011) and this may indicate that, compared to VWM behavioral estimates, CDA is more sensitive to resolution variations according to saliency.

### Other Socially Relevant Factors and Interindividual Differences

Other investigations combined different emotional stimuli for understanding how social information is integrated into VWM. Negative emotional words presented during the retention interval (2,000 ms) seem to enhance performance (*d’*) for angry faces (compared to happy) (Jackson et al., 2014). An angry benefit emerged with both positive and negative words when using a longer retention interval (9,000 ms; Jackson et al., 2012). Authors suggested that encoding negative faces creates a condition (*threat tagging*) in which identity is coupled with valence and congruent stimuli (i.e., negative words) can interact with this representation (Jackson et al., 2014). Maran et al. (2015) induced positive or negative mood using high-impact pictures (e.g., erotic, mutilations, etc.) and observed improved performance (*d’*) for all emotional faces. Similarly, inducing a feeling of social exclusion (Du et al., 2019) or including a monetary reward (instead of penalty; Thomas et al., 2016) improved VWM capacity for faces. On the contrary, a facial task during the retention interval while maintaining a face in VWM seems to decrease accuracy (Robinson et al., 2008). Overall, VWM for faces seems to benefit from non-facial emotional stimuli such as words or other non-visual factors (i.e., mood).

Dealing with task-relevant and irrelevant (distractors) information is another important VWM facet. Filtering efficiency interacts with individual VWM capacity (Vogel et al., 2005) and with psychopathology (Stout et al., 2013, 2015). CDA seems to be an optimal measure for this purpose. Given the correlation with the number of to-be-memorized items until capacity limit (Vogel and Machizawa, 2004), CDA amplitude for *n* task-relevant stimuli should be greater than amplitude for *n* stimuli, some of which are task-irrelevant. Including emotional face distractors in the memory array (happy, angry, and neutral) and using an identity CDT (1 or 2 to-be-remembered faces), Ye et al. (2018) found that high-capacity subjects filtered out all distractors compared to low-capacity subjects in whom filtering activity was effective only for happy faces.

Psychopathology is another critical factor in social cognition. Anxiety, in particular, has been widely studied in relation to WM and generally correlates with lower WM capacity (for a review, see Moran, 2016). In two different experiments using a location probe task with real emotional faces (angry, neutral, and happy), Yao et al. (2018) demonstrated lower VWM capacity (Cowan’s *K*) for all facial expressions in individuals with higher self-reported anxiety, without affecting precision.

Filtering irrelevant information is an important WM function and could be relevant in anxiety (Qi et al., 2014). Using an identity CDT and monitoring the CDA, Stout et al. (2013) measured the filtering efficiency for task-irrelevant faces (with fearful or neutral expressions). They found that task-irrelevant fearful faces were less efficiently filtered out compared to neutral faces. In addition, filtering efficiency negatively correlated with self-reported anxiety. More specifically, Stout et al. (2015) demonstrated that filtering efficiency is specifically inversely related to the worry component of anxiety. Moreover, Meconi et al. (2013) using an identity CDT reported greater CDA amplitude for trustworthy faces. Interestingly, when self-reported anxiety was considered, untrustworthy faces (vs. trustworthy) were associated with larger CDA amplitude in the most anxious participants.

Other clinical conditions have been studied in relation to facial expression VWM representations. Patients with schizophrenia seem to have an overall WM deficit (Forbes et al., 2009) and lower VWM capacity for neutral faces (She et al., 2019). Interestingly, using emotional faces, the angry benefit is still present although an emotion classification deficit is observed (Linden et al., 2010). Individuals with melancholic depression have a VWM bias (i.e., higher *d*’) toward sad faces compared to individuals with non-melancholic depression (Linden et al., 2011). In an expression change localization task, individuals with high suicidal intentions seem to have worse VWM capacity for negative schematic faces compared to controls (Xie et al., 2018). Furthermore, Takahashi et al. (2015) using a CDT with schematic faces (angry, happy, and neutral) demonstrated that high alexithymic individuals have worse VWM capacity for happy faces compared to low alexithymic individuals.

## Discussion and Conclusion

Faces are complex stimuli that convey multiple information and that seem to be subject to a special type of holistic processing during early stages of processing. For this reason, it is plausible to hypothesize that faces are also represented in VWM in a “special” way when compared to non-facial stimuli or inverted faces. Many of the studies in the literature have focused on the effects of facial expressions of emotions (both task-relevant with schematic faces, and task-irrelevant with real faces of different identities) on the representation of faces in VWM. Negative faces, in particular angry, are associated with better VWM performances. However, a great methodological variability in stimuli choice and CDT parameters makes it difficult to compare findings. As previously shown, results could drastically change using schematic vs. real faces or different probing methods. Future research in this field, if not of interest, should keep paradigms’ parameters fixed, only varying socially relevant information. Otherwise, an orthogonal variation of CDT parameters within the same study could be useful (e.g., using several encoding times, schematic vs. real faces).

VWM is defined a hub of cognition (Luck, 2008) where information is retained and manipulated. Interestingly, different socially relevant information (e.g., emotional words or mood) seems to interact with facial memory representations. Ecologically, integrating different sources of social information could be an adaptive mechanism.

Psychopathology is another important aspect in social environment and often related to changes in basic cognitive functions. Again, different methods and different psychopathological conditions are difficult to integrate. However, it is interesting noting that psychopathology and VWM functioning are related. Alexithymic individuals have the worst VWM performance for happy faces (Takahashi et al., 2015) and individuals with suicidal intentions show the worst VWM performance for negative stimuli, probably originating from an adaptive avoidance behavior (Xie et al., 2018).

At the neural level, the CDA seems to be influenced by facial information. It has been demonstrated that the CDA is modulated according to the amount (Vogel and Machizawa, 2004) and also the quality (i.e., resolution) of visual information (Luria et al., 2016). Interestingly, even with a single to-be-remembered face (i.e., capacity estimation is not relevant), the CDA is modulated by facial information (Sessa et al., 2011, 2018; Meconi et al., 2013). According to *flexible resource models* and the *neural object-file theory* (Xu and Chun, 2006, 2009), one important and ecologically relevant aspect to be considered could be the resolution variation according to saliency. The theory proposes two stages of processing (with neural bases on distinct part of IPS that is supposed to be also the CDA generator), where the second stage regards a detailed visual encoding of relevant objects. Integrating this neural measure in standard behavioral studies and focusing on resolution besides capacity could be useful for finely comparing representations of different socially relevant information.

## Author Contributions

FG wrote the first draft of the manuscript. PS provided critical revision. Both authors read and approved the submitted version.

### Conflict of Interest

The authors declare that the research was conducted in the absence of any commercial or financial relationships that could be construed as a potential conflict of interest.
